# Successful eradication of renal allograft abscess by CT-guided percutaneous pigtail drainage: A case report

**DOI:** 10.1097/MD.0000000000033551

**Published:** 2023-04-14

**Authors:** Jing Gang Ding, Ge Zhang, YuHui Wang

**Affiliations:** a Department of Critical Care Medicine, Sir Run Run Shaw Hospital Zhejiang University School of Medicine, Hang Zhou, Zhe Jiang, China; b Department of Critical Care Medicine, Sir Run Run Shaw Hospital Zhejiang University School of Medicine, Hang Zhou, Zhe Jiang, China.

**Keywords:** case report, percutaneous pigtail drainage, polymicrobial abscess, renal allograft abscess

## Abstract

Renal allograft abscess is an infrequent complication in kidney transplant recipients. The mainstay of treatment is adequate drainage and optimal antibiotic administration.

**Patient Concerns:**

A 25-year-old female who presented with dysuria, frequency, chills, fever, and allograft pain was initially diagnosed with a urinary tract infection complicated by septic shock 15 months after kidney transplantation. Ultrasound depicted a hypoechoic mass and contrast-enhanced computed tomography (CT) revealed a lesion with no enhancement in the renal allograft. CT-guided percutaneous pigtail drainage placement was implemented.

**Diagnoses:**

Blood-stained pus was aspirated from the lesion in the renal allograft. The aspirate culture revealed *Escherichia coli* and Proteus mirabilis with an antibiogram consistent to urine culture. The diagnosis of renal allograft abscess originated from urinary tract infection was confirmed.

**Interventions:**

The patient underwent CT-guided percutaneous pigtail drainage and conducted culture of the aspirate.

**Outcomes:**

The patient’s symptoms immediately abated after drainage and renal allograft function recovered normally. Ultrasound and CT showed total regression of the renal allograft abscess at the 1-month outpatient follow-up.

**Lessons:**

Heightened alertness should be attached to that severe urinary tract infections presenting with sepsis shock and antibiotic treatment non responders are likely to progress to renal allograft abscess in kidney transplant recipients. CT-guided percutaneous pigtail drainage was a safe and effective minimally invasive treatment.

## 1. Introduction

Renal and perirenal abscesses are an infrequent infectious complication in kidney transplant recipients.^[1]^ Furthermore, diagnosis of them is still a challenge since the symptoms can be insidious and obscure.^[2]^ Delayed diagnosis may lead to higher morbidity and mortality.^[3]^ Ultrasound and computed tomography (CT) do not only provide early diagnosis but also guide percutaneous therapeutic interventions.^[4]^ Ultrasound- or CT-guided percutaneous drainage is minimally invasive procedure associated with a high rate of success and a low complication rate.^[5]^ Herein, we report the first case of a polymicrobial abscess caused by *Escherichia coli* and Proteus mirabilis in the renal allograft. CT-guided percutaneous pigtail drainage (PPD) combined with antibiotics resulted in complete resolution.

## 2. Case report

A 25-year-old unmarried female was treated with peritoneal dialysis for 2 months due to end-stage renal failure of unknown etiology and underwent a deceased donor kidney transplantation 15 months ago. She was then treated with induction immunosuppressive therapy included basiliximab, mycophenolate mofetil and steroids, and maintenance immunosuppression with tacrolimus, mycophenolate mofetil and steroids. She was complicated with a urinary tract infection (UTI) postoperative 4 months and *E coli* producing extended spectrum beta lactamase (ESBL) were cultured in urine. A short course of intravenous imipenem was initiated showing a good response.

**Figure 1.**
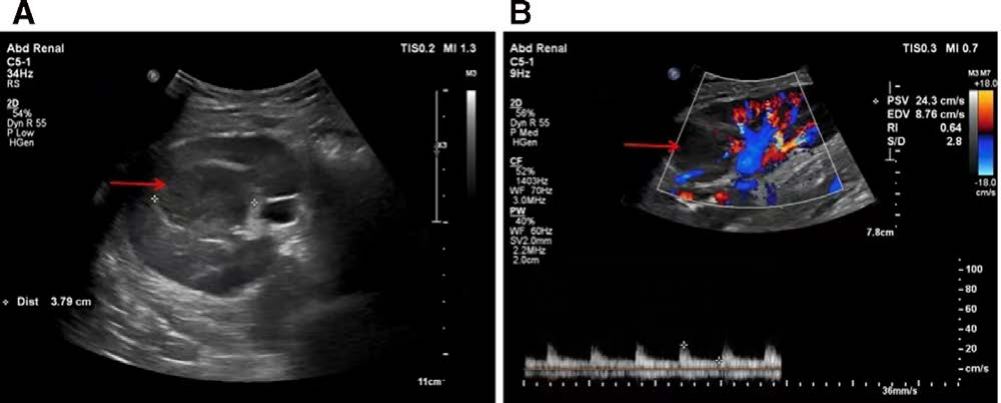
(A–B) Color Doppler ultrasound depicted a hypoechoic lesion of 4.3 × 3.8cm with no blood flow signals inside in the renal graft (arrow).

**Figure 2.**
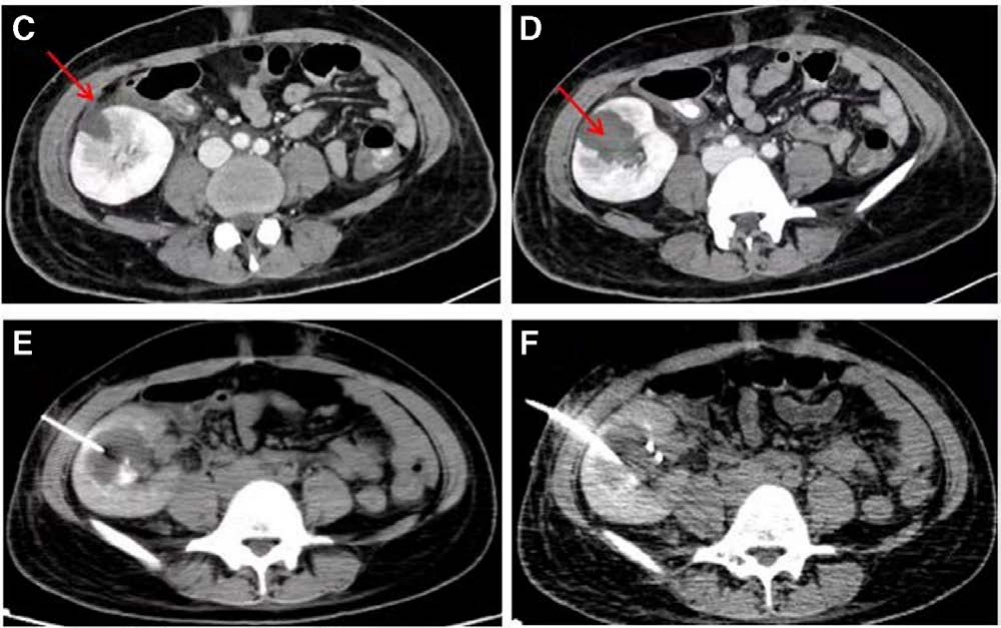
(C–F) Contrast-enhanced CT revealed a lesion with no enhancement indicative of abscess formation, which involved renal parenchyma and medulla and ruptured into perirenal space (arrow). CT = computed tomography.

She presented to the fever clinic with dysuria and frequency, followed by chills, fever, and allograft pain 2 days ago. Her temperature was 39.5°C. Physical examination revealed renal allograft tenderness. The laboratory investigations revealed a white blood cell (WBC) count of 8 × 10^3^/µL with 87.3% neutrophils. Urinalysis by dipstick detected the presence of leukocyte esterase and nitrite. Serum C-reactive protein, procalcitonin (PCT), liver and kidney function parameters were normal. Chest and abdomen CT scans showed no abnormality. She was treated with oral levofloxacin The next day, she presented to the emergency department with nausea, vomiting, fatigue, dizziness, oliguria and hypotension of 86/49 mm Hg. Arterial blood gas analysis indicated elevated lactate of 2.9 mmol/L (reference range 0.5–2.2 mmol/L). WBC count, C-reactive protein, PCT and creatinine were elevated (Table 1). She was admitted to intensive care unit due to severe infection. Despite adequate fluid resuscitation, she presented with hemodynamic instability and arterial blood gas analysis still showed elevated lactate of 8.0 mmol/L. Thus, norepinephrine were on. Bedside renal ultrasound found a well-bounded hyper-echogenic mass of 4.0 × 3.8 cm in the upper and middle pole of renal allograft and blood flow signals were visible inside on color Droppler (the images were not saved). Given a recent history of *E coli* (ESBL+) infection and renal allograft dysfunction, the empirical broad-spectrum intravenous antibiotics with linezolid and imipenem were administered. The immunosuppressive agents simultaneously were decreased. Tracheal intubation and mechanically assisted ventilation were performed for respiratory distress due to pulmonary edema. She had chills and fever lasting more than 72 hours and WBC count, CPR, and PCT were still rising (Table 1) in spite of adequate antibiotics. Repeated color Doppler ultrasound depicted a hypoechoic lesion of 4.3 × 3.8 cm with no blood flow signals inside in the renal allograft (Fig. 1). A subsequent contrast-enhanced CT revealed a lesion with no enhancement indicative of cortico-medullary abscess formation extending to perirenal space. A 18G needle was inserted into the lesion under CT guidance and 8F pigtail catheter (ARGON MEDICAL DEVICES INC, Athens, America) placement was performed (Fig. 2) and blood-stained pus was aspirated. Her symptoms improved immediately and inflammatory parameters decreased significantly after drainage, fever and renal allograft pain disappeared, shock was corrected, endotracheal intubation was successfully removed. The Urine culture yielded *E coli* (ESBL−)>10^5 cfu/mL and Proteus mirabilis > 10^5 cfu/mL; the blood culture isolated *E coli*; the aspirate culture revealed *E coli* and Proteus mirabilis. And their antibiotic sensitivity tests were consistent. So targeted antibacterial therapy with ertapenem was provided. After a total 2-week course of intravenous antibiotic therapy and the resolution of signs and symptoms, the antibiotic regimen was switched to oral levofloxacin. She was discharged with complete improvement of clinical and biological parameters on post-drainage day 22. Repeat ultrasound and CT showed total regression of the abscess at the 1-month follow-up.

**Table 1:** Blood analysis.

Variables	value				
	FC	ED	ICU-d1	ICU-d3†	ICU-d6
WBC (3.5-9.5 × 109/L)*	8	10	11	26.2	3.3
CRP (<6mg/L) *	5.2	38.8	77.3	291.5	27.7
PCT (<0.05ng/mL) *	<0.04	15.42	34.36	49.49	13.96
Creatinine (41–73μmol/L) *	77	155	158	130	78

CRP = C-reactive protein, ED = emergency department, FC = fever clinic, ICU = intensive care unit, PCT = procalcitonin, WBC = white blood cell.

* Reference values in parentheses.

† The day of CT-guided pigtail catheter placement was performed.

## 3. Discussion

Renal and perirenal abscesses are a comparatively rare complication of UTIs.^[6]^ Immunosuppression, diabetes mellitus and obstructive uropathy are predisposing risk factors.^[7]^ However, renal and perirenal abscesses can occur in an otherwise normal urinary tract.^[8]^ kidney transplant recipients in immunocompromised state are at elevated risk of severe and life threatening conditions.^[9]^ *E coli*, Proteus mirabilis and S. Aureus are the most frequently causative organisms.^[7]^ The incidence of polymicrobial abscess has been increasingly frequent, ranging from 19.2% to 33.3%.^[3,10]^

Imaging is not routinely indicated in uncomplicated UTIs and renal infections which are usually diagnosed based on clinical symptoms and laboratory data.^[9]^ However, imaging of the genitourinary tract is warranted in cases of severe infection to exclude progression to renal or perirenal abscess.^[11]^ Moreover, imaging plays a crucial role in evaluating renal inflammatory lesions under specific situations like immunocompromised hosts, treatment non responders, equivocal clinical diagnosis and post-transplant for evaluating extent of disease.^[9]^ Ultrasound is usually the initial imaging modality due to its safety, low cost and availability for serial examinations.^[12]^ However, ultrasound is not sensitive to identify small abscesses since smaller abscess size contributes to misdiagnosis.^[3]^ In some cases, the abscess may present as an echogenic mass which is difficult to recognize by ultrasound, so that contrast-enhanced CT is essential for the definite diagnosis.^[6]^ CT can show fluid collections and their anatomic relationship to adjacent structures superior to ultrasound can.^[13]^ Thereby, CT is more sensitive than ultrasound for assessing abscess and its spread.

The backbone of treatment for renal or perirenal abscess is adequate drainage and optimal antibiotic regimens.^[3]^ The choice of antibiotics ideally should be based on culture results; however, this is inevitably delayed in acquiring results.^[2]^ Empirical broad-spectrum intravenous antibiotics should be initiated. Once susceptibility data are available, targeted antibiotic regimens should be prescribed accordingly.^[2]^ There is no consensus on optimal duration of renal or perirenal abscess, which should be longer than the course of complicated UTIs with 14 to 21 days and extended until adequate drainage of abscesses has been achieved.^[11]^

For small renal abscesses <3cm in diameter may resolve with antibiotic treatment alone. Otherwise, ultrasound- or CT-guided percutaneous aspiration and drainage is indicated for treatment combined with antibiotics. Although, Lee et al^[14]^ reported a high clinical success rate of 96.08% (49/51 abscesses measuring 5 cm or less) using intravenous antibiotics alone, they could not compare the outcome of antibiotics treatment alone to that of percutaneous drainage. Intravenous antibiotic therapy, indeed, can be feasible in selected cases when therapeutic drainage implies a considerable risk.^[6]^ Thanks to the recent development of interventional techniques, renal and perirenal abscesses can be managed sucessfully by ultrasound- or CT-guided minimally invasive percutaneous procedures, which enable to avoid open surgical drainage, nephrectomy or nephrostomy tube placement. Ultrasound- or CT-guided percutaneous catheter drainage, performed under local anesthesia, was accepted as the primary method of treatment in view of its low complication and mortality rate and high patient tolerability.^[4]^ A recent research reported that the clinical success rate was 100% for renal-perirenal abscesses.^[4]^

The initial diagnosis of our patient was a complicated UTI based on clinical symptoms and laboratory data. The initial abdomen CT scans found no abnormalities including urinary obstruction and urinary calculi. The patient’s illness rapidly deteriorated into septic shock. The initial ultrasound revealed a well-bounded hyper-echogenic mass in the renal allograft. However, we did not identified it as an abscess. Although intensive antimicrobial therapy was rendered and the immunosuppressive agents were decreased, the patient’s condition did not improve. The repeat ultrasound and contrast-enhanced CT prompted the abscess formation and CT-guided PPD placement was subsequently carried out. After that, the patient’s symptoms immediately improved and the abscess was ultimately completely resolved. Here, we report a case of renal allograft abscess complicated with bacteremia and septic shock, which was successfully eradicated via CT-guided PPD and antibiotics. The abscess was originated from UTI in an otherwise normal urinary tract due to the same pathogenic bacteria and antibiotic sensitivity tests. With this report, we hope to highlight several features conducive to the successful clinical management of renal allograft abscess, including; Kidney transplant recipients in immunocompromised state are prone to developed severe UTIs complicated by sepsis or septic shock. UTI-related renal allograft abscess should be suspected as early as possible even if the urethra is normal; Diagnostic imaging such as ultrasound and CT especially contrast-enhanced CT promptly recognize the renal allograft abscess when antibiotic treatment non responders, serial evaluation sometimes is needed; CT-guided PPD is a minimally invasive access to the abscess, which provides the knowledge of the underlying organisms and their antimicrobial susceptibilities and directs targeted treatment. Overall, we believe CT-guided PPD stands out as a safe and efficient minimally invasive approach to the renal allograft abscess, which allows a promising clinical outcome.

## Author contributions

**Resources:** YuHui Wang.

**Writing – original draft:** Jing Gang Ding.

**Writing – review & editing:** Ge Zhang.

## References

[1] Shoja MM Ardalan MR Etemadi J. Renal allograft abscesses following transplant: case report and literature review. Exp Clin Transplant. 2007;5:720–3. 18194131

[2] Liu XQ Wang C-C Liu Y-B. Renal and perinephric abscesses in West China Hospital: 10-year retrospective-descriptive study. World J Nephrol. 2016;5:108–14. 26788470 10.5527/wjn.v5.i1.108 PMC4707163

[3] Lin HS Ye J-J Huang T-Y. Characteristics and factors influencing treatment outcome of renal and perinephric abscess–a 5-year experience at a tertiary teaching hospital in Taiwan. J Microbiol Immunol Infect. 2008;41:342–50. 18787743

[4] Yildirim G Karakas H. Percutaneous catheter drainage in retroperitoneal abscesses: a single centre’s 8-year experience. Pol J Radiol. 2022;87:e238–45. 35582601 10.5114/pjr.2022.115815 PMC9093211

[5] Kobayashi K Censullo ML Rossman LL. Interventional radiologic management of renal transplant dysfunction: indications, limitations, and technical considerations. Radiographics. 2007;27:1109–30. 17620470 10.1148/rg.274065135

[6] Trumm CG Burgard C Deger C. Intermittent quick-check CT fluoroscopy-guided percutaneous drainage placement in patients with infected renal and perirenal fluid collections: 11-year experience. Diagn Interv Radiol. 2021;27:378–85. 34003125 10.5152/dir.2021.20068 PMC8136537

[7] Patino A Martinez-Salazar EL Tran J. Review of imaging findings in urinary tract infections. Semin Ultrasound CT MR. 2020;41:99–105. 31964498 10.1053/j.sult.2019.09.004

[8] Shu T Green JM Orihuela E. Renal and perirenal abscesses in patients with otherwise anatomically normal urinary tracts. J Urol. 2004;172:148–50. 15201757 10.1097/01.ju.0000132140.48587.b8

[9] Das CJ Ahmad Z Sharma S. Multimodality imaging of renal inflammatory lesions. World J Radiol. 2014;6:865–73. 25431641 10.4329/wjr.v6.i11.865 PMC4241493

[10] Fulla J Storme O Fica A. [Renal and perinephric abscesses: a series of 44 cases]. Rev Chilena Infectol. 2009;26:445–51. 19915755

[11] Goldman JD Julian K. Urinary tract infections in solid organ transplant recipients: guidelines from the American society of transplantation infectious diseases community of practice. Clin Transplant. 2019;33:e13507. 30793386 10.1111/ctr.13507

[12] Tezcan S. Ultrasonography findings of urinary tract infection after kidney transplant: a case report. Exp Clin Transplant. 2018;16 Suppl 1(Suppl 1):119–21. 10.6002/ect.TOND-TDTD2017.P22 29528007

[13] Inci MF Ozkan F See TC. Renal transplant complications: diagnostic and therapeutic role of radiology. Can Assoc Radiol J. 2014;65:242–52. 24325923 10.1016/j.carj.2013.06.002

[14] Lee SH Jung HJ Mah SY. Renal abscesses measuring 5 cm or less: outcome of medical treatment without therapeutic drainage. Yonsei Med J. 2010;51:569–73. 20499424 10.3349/ymj.2010.51.4.569 PMC2880271

